# An engineered biofactory for efficient production of diverse recombinant superoxide dismutase isozymes loaded with specific metal ions for biochemical characterisation

**DOI:** 10.64898/2026.07.08.737244

**Published:** 2026-07-09

**Authors:** Rafał Mazgaj, Agnieszka Kołpa, Mariam Esmaeeli, Justyna Pełczyńska, Diana Galea, Jan Gawor, Agata Malinowska, Agnieszka Szczypiorowska, Thomas Kehl-Fie, Kevin J. Waldron

## Abstract

**Background:**

Biochemical, biophysical and structural characterisation of isozymes from the ubiquitous family of iron- or manganese-dependent superoxide dismutases (SodFMs) requires the purification of high-quality preparations of recombinant enzymes. Determination of their key biochemical parameter, their catalytic metal-preference, requires the comparison of the catalytic turnover of samples loaded exclusively with iron versus samples loaded exclusively with manganese. Both of these aims are inhibited by the potential contamination of recombinant preparations of SodFMs, prepared by heterologous overexpression inside *Escherichia coli* cells, by even low levels of endogenous SodFMs from the host, both of which show very high turnover with either manganese ( *E. coli* MnSOD) or iron (FeSOD). To overcome this problem, we created a strain of *E. coli* lacking the endogenous SodFMs. Here, we characterised this *E. coli* BL21 (DE3) Δ *sodA* Δ *sodB* strain, determining the physiological effects of SodFM deletion and demonstrating its utility for producing recombinant SodFMs for *in vitro* characterisation and use.

**Results:**

Genomic analysis verified the targeted gene deletions, without off-target effects. Growth, expression, elemental analysis, and proteomic data confirmed a lack of physiological defects of the strain except for a known inability to grow on glucose, which is overcome by heterologous SodFM expression. We demonstrate the utility of the strain for the efficient production of diverse recombinant SodFMs, including highly divergent, understudied isozymes, including the ability to precisely control the metal-loading of the heterologously expressed protein.

**Conclusions:**

The *E. coli* strain described herein is a useful microbial cell factory for production of recombinant SodFMs, which should find widespread utility as expression host of choice, enabling more efficient production of protein for studies of the biochemical, biophysical and structural properties of this remarkable family of metalloenzymes.

## Full Text Availability

The license terms selected by the author(s) for this preprint version do not permit archiving in PMC. The full text is available from the preprint server.

